# Miscibility and Optimization of the Liquid Rubber Content in the Resins of Light-Cured Dental Composites

**DOI:** 10.3390/ma16010087

**Published:** 2022-12-22

**Authors:** Krzysztof Pałka, Monika Sowa

**Affiliations:** Faculty of Mechanical Engineering, Lublin University of Technology, Nadbystrzycka 36, 20-618 Lublin, Poland

**Keywords:** liquid rubber, dimetacrylate resins, dental composite, miscibility, mechanical properties

## Abstract

Fracture toughness is one of the main factors influencing the durability of light-cured composites used for dental restorations and fillings. One of the methods of increasing the fracture toughness is the modification of the matrix with liquid acrylonitrile-free liquid rubber. This study aimed to assess the miscibility of acrylonitrile-free liquid rubber with a blend of resins and their stability over time, and to determine the optimal amount of liquid rubber (LR) in the blend due to mechanical properties. Two blends of dimethacrylate resins were used: resin “F” composed of BisGMA (60 wt.%), TEGDMA (20 wt.%), BisEMA (10 wt.%) and UDMA (10 wt.%), and “C” resin containing BisGMA (40 wt.%), TEGDMA (40 wt.%), BisEMA (10 wt.%) and UDMA (10 wt.%). The modifier Hypro^®^ 2000X168LC VTB liquid rubber was used in at 1%, 2%, 3%, 4%, 5%, 10%, 15% and 20% by weight in the resin blend. The miscibility was assessed by microscopy. The fracture toughness, flexural strength and Young’s modulus were determined in the bending test. The results showed that the solubility of the liquid rubber depends on the ratio of BisGMA/TEGDMA in the resins. In resins with 40 wt.% TEGDMA, the LR solubility was as high as 5%, while resins with 20 wt.% TEGDMA, the liquid rubber did not dissolve. The LR-resin mixtures showed good time stability, and no changes in the size or morphology of the rubber domains were found after 24 h of mixing. The maximum fracture toughness (2.46 MPa m^1/2^) was obtained for 5 wt.% LR in resin F and for 15 wt.% LR in resin C (2.53 MPa m^1/2^). The modification with liquid rubber resulted in an exponential reduction in both flexural strength and Young’s modulus. The analysis of the results of the mechanical tests allowed us to determine the optimal amount of LR for both resins. For resin F it was 5.4 wt.%, and for resin C it was 8.3 wt.%. It can be stated that the optimal amount of liquid rubber increases with its solubility in the resin.

## 1. Introduction

Light-cured dental composites have been developed for over 50 years, and the main focus has been on changing the reinforcement while the resin matrix has not been developed much. Until the mid-1990s, nearly all commercial composites contained BisGMA resin. Other monomers used are mainly TEGDMA, UDMA and BisEMA, while other methacrylates or other types of monomers (tricyclodecane or linear polyurethane) have rarely been used [1]. Dimethacrylates, like many other resins, inherently show low fracture toughness [2]. This resulted in a significant amount of damages to these fillings and restorations at the beginning of the use of composite materials in dentistry. The damage rate of the fillings was as high as 50% after 10 years [3]. Thanks to the continuous improvement of materials, a significant increase in the durability of fillings has been achieved. Currently, the most favored are hybrid composites, for which the annual failure rate is 1.5–2%, usually as a result of fractures [4].

Improvement of the fracture toughness of composites can be obtained by modifying the matrix with an elastomeric soft phase. This phase is usually liquid rubber or rubber, and the strengthening mechanisms of resins using an elastic phase is called rubber toughening (RT) or rubber modification [2]. Modifying the matrix of dental composites can not only increase fracture toughness but also reduce polymerization shrinkage [5] while increasing the conversion [6] and adhesion to tooth tissues [7]. The problem of modifying the resins with liquid rubber has been broadly described for epoxy resin [2,8,9], while only a few studies focused on the modification of dimethacrylate resins [10].

One strategy to modify the resins with an elastic phase is to use pre-formed elastomeric particles [11]. Structural core-shell particles, (CSP or core-shell rubber, CSR) with a rubber core and a thin layer of hard shell are mostly used. Importantly, the particle size and volume fraction can be easily controlled. The coating of the particles protects against the formation of agglomerates and changes in the size and shape of the particles, and facilitates the performance of technological processes. The core-shell structure makes it possible to independently change the composition of the core and the shell. The core in such modifiers is usually made of acrylic or butadiene-based rubber. The role of the coating is to chemically adjust the shell to the matrix material for proper bonding [2]. The mechanism responsible for increasing the fracture energy in such materials has been described as particle cavitation and energy absorption during plastic shear of the matrix. However, this form of reinforcement can affect the shade of the filling and cause problems with polishing.

In the second strategy to toughen resins with elastomers, reactive compounds that are miscible or soluble in the resin (e.g., liquid rubber) are used [10]. After initiating the curing reaction and the molecular weight begins to increase, the second phase is separated in the form of rubber particles [12]. The term “liquid rubber” is commonly used to refer to elastomers that are viscous enough and have a low enough molecular weight to allow homogeneous mixing within the resin. The most commonly used components are liquid compounds based on polybutadiene or acrylonitrile-polybutadiene copolymers with different acrylonitrile content. To improve the interfacial adhesion to the polymer matrix, these elastomers are usually terminated with functional groups capable of reacting with the resin. The most common modification is a terminal carboxyl group, producing carboxyl-terminated butadiene (CTB) or carboxyl-terminated butadiene-acrylonitrile (CTBN) with a different acrylonitrile (AN) content [11]. Amine-modified (ATB/ATBN), epoxy (ETB/ETBN) or vinyl-modified (VTB/VTBN) liquid rubbers are also available [13]. Unfortunately, rubbers containing acrylonitrile have the potential to cause carcinogenicity, which may limit the use of these materials [14].

In the field of modifying resins with liquid rubber in dental applications, the first attempts used low-molecular-weight modifiers based on butadiene and styrene-butadiene to improve the fracture toughness of the resins used as prosthetic bases [15,16,17,18]. These are also described in patent US3427274 [19]. Rodford [17] modified PMMA with low-molecular-weight styrene-butadiene rubber. Such modification reduced the value of Young’s modulus by as much as 20%, while the fracture toughness increased by over 80% in the range of rubber concentrations of 6–15% (wt./vol.). Kerby et al. [10] used poly (butadiene-acrylonitrile-acrylic acid) (PBDM) terpolymers with methacrylate ends (4:1 ratio of butadiene to acrylonitrile) to modify the mixture of BisGMA and TEGDMA resins (50 wt.% each). The results showed much better fracture toughness, especially with a PBDM content of 10%, and reduced water sorption. These results, however, can only be applied to the modification of a specific mixture of BisGMA and TEGDMA as TEGDMA usually acts as a solvent for high-viscosity resins [17].

The purpose of this work was to determine the optimal amount of LR in the blend because of its mechanical properties, as well as to assess the miscibility of the liquid acrylonitrile-free LR with the blends. The null hypothesis is there will be no difference between resin blends without and after modification with liquid rubber. 

## 2. Materials and Methods

### 2.1. Materials

Two resin blends that can be used in commercial materials were used in the following mixtures with their designations:

- resin *F* (intended for flow-type material), composed of BisGMA (60 wt.%), TEGDMA (20 wt.%), BisEMA (10 wt.%) and UDMA (10% wt.)
- resin *C* (intended for conventional composite), composed of BisGMA (40 wt.%), TEGDMA (40 wt.%), BisEMA (10 wt.%) and UDMA (10 wt.%)

The mixture was supplemented in each case with 1% by weight of photoinitiator (camphorquinone), co-initiator (2-dimethylaminoethyl methacrylate, DMAEMA) and an inhibitor (butylated hydroxytoluene, BHT). All resins and additives were purchased from Sigma-Aldrich Chemicals (Munich, Germany). 

The resin used in the production of the flow composite (resin F) had a higher viscosity because less reinforcement could be introduced into it (approx. 50% by weight). Resin C, on the other hand, must absorb a higher amount of reinforcement (up to 80% by weight), and hence, a lower viscosity is necessary in this case. The viscosity was assessed organoleptically under the same temperature conditions. 

Preparation of the resin blends with liquid rubber was partially based on the patent [20]. The resin blends were mechanically mixed at the Arkona Laboratory of Dental Pharmacology until a homogeneous mixture was obtained (1 h). The blends were then mixed mechanically (1 h) with Hypro^®^ 2000X168LC VTB liquid nitrile-free rubber (Huntsman International LLC, Salt Lake City, UT, USA) to obtain the following LR concentrations (by weight): 1%, 2%, 3%, 4%, 5%, 10%, 15% and 20%. The blends with LR concentrations in the range of 1% to 4% were used only for the miscibility assessment. Mixing was performed in a yellow-light environment to prevent the materials from curing.

### 2.2. Miscibility of Resin Blends and Liquid Rubber

The miscibility and time stability were assessed by light optical microscopy (LOM) (Eclipse MA200, Nikon, Tokyo, Japan) with the use of an external illuminator with yellow light. A drop of material was applied to the slide immediately after mixing the resin with the rubber and the first images were acquired. The samples were placed on a microscope base slide with spacers placed at its edges to ensure a resin layer thickness of 20 µm. After covering the sample with a coverslip, the desired thickness was achieved. Subsequent images were acquired after 1 and 24 h, preventing the material from polymerization using a non-translucent cover. The final images were taken after curing.

### 2.3. Specimens Preparation

Samples were prepared for fracture toughness tests based on the ASTM E 399-20 standard [21]. According to the mentioned standard, the transverse dimensions of the samples should meet the condition 1 ≤ *W/B* ≤ 4, where *W* is the height and *B* is the width of the sample. Moreover, the spacing of the bending supports should be *L =* 4·W. The notch depth *a* measured after specimen fracture, should be within the limits *a* = 0.45–0.55 W. The angle at the notch point should be less than 90°. Following the published literature [21,22], the following dimensions of the samples were established: 15 mm × 2.2 mm × 2.2 mm (length, height and width, respectively), and the support span was *L* = 8.8 mm, and the notch angle was 80°.

The specimens for the flexural test were prepared in accordance with ISO 4049 using specimens with dimensions of 25 mm × 2 mm × 2 mm (length × height × width, respectively), while the support span *L* was 20 mm.

As the test material is liquid, the samples were made by the casting method. For this purpose, model plates were prepared with three sample models each. One sample was used for fracture toughness testing, while the other samples were used for bending. The plates were modeled in Solid Edge 2021 and made on a CNC machine using EN-AW 2024 aluminum alloy.

**Figure 1:**
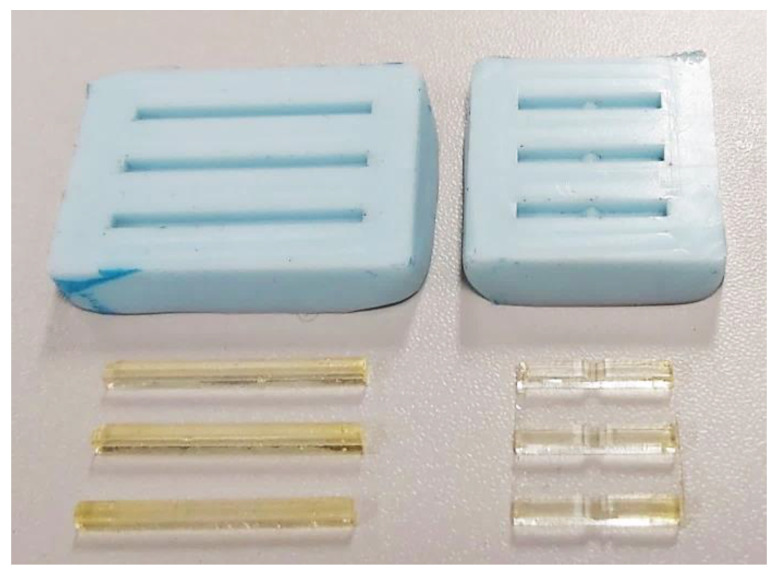
Molds and manufactured samples.

The mold for sample manufacturing (Figure 1) was made by pouring a two-component Siliform series casting silicone (Dreve Dentamid, Unna, Germany) on the model plates. Siliform is characterized by a high degree of accuracy in reproduction, high dimensional stability over time and its shrinkage is 0.3%, which guaranteed the desired dimensions of the samples.

The resin blends and their mixtures with LR were poured into the molds, covered with a Mylar-type film and cured with a LED lamp of intensity 1350 mW/cm^2^ and wavelength of 440–480 nm for 20 s over each area, starting from the center of the sample and then moving the optical fiber every 8 mm to the left and then right from the center so that the entire surface was exposed. The optical fiber had a diameter of 10 mm, so an overlap of the irradiated areas was obtained. The 15 mm long samples were irradiated in three places, while the 25 mm samples were irradiated in five places. The distance between the fiber end and the sample was the same each time because of the Mylar-type foil. This method of shifting the optical fiber complies with the ISO 4049 recommendations. After preparation, the samples were stored for 24 h in distilled water at 37 °C until testing.

### 2.4. Mechanical Properties

#### 2.4.1. Fracture Toughness

Fracture toughness *K_Ic_* was determined according to the ASTM E 399-20 [21] standard, using notched bending specimens. The tests were carried out on an Autograph AG-X Plus (Shimadzu, Kyoto, Japan) testing machine at a traverse speed of 0.5 mm/min, to measure the maximum force *P* at the sample fracture. The value of *K_Ic_* [MPa m^1/2^] was determined according to the formula [21,22]:

(1) $$K_{Ic}=\frac{3\sqrt{\frac{a}{W}}\left[1.99-\frac{a}{W}\left(1-\frac{a}{W}\right)\left(2.15-3.93\frac{a}{W}+2.7{\left(\frac{a}{W}\right)}^{2}\right)\right]PL}{2\left(1+2\frac{a}{W}\right){\left(1-\frac{a}{W}\right)}^{\frac{3}{2}}BW^{\frac{3}{2}}}$$

Ref. [23] provides other equations to determine the fracture toughness, but the results obtained with them are very similar to those from Equation (1).

#### 2.4.2. Flexural Strength

The flexural strength *σ_f_* (MPa) was determined in a three-point bending test according to the ISO 4049 standard [24]. The tests were carried out on the Autograph AG-X Plus (Shimadzu, Kyoto, Japan) testing machine at a traverse speed of 0.5 mm/min.

The flexural strength was calculated according to the formula:

(2) $$\sigma_{f}=\frac{3PL}{2BW^{2}}$$

#### 2.4.3. Young’s Modulus

Young’s modulus, *E* (GPa), was calculated based on the EN ISO 178 standard using data from the flexural strength test according to the formula:

(3) $$E=\frac{\sigma_{f2}-\sigma_{f1}}{\varepsilon_{f2}-\varepsilon_{f1}}$$

where: ε*_f_*_1_ = 0.0005, *ε_f2_* = 0.0025, and *σ_f_*_1_ i *σ_f_*_2_ are the stresses calculated for strains *ε_f_*_1_ i *ε_f_*_2_, respectively.

#### 2.4.4. SEM Imaging

After the mechanical tests, the fractures of the samples were observed using a Nova NanoSEM 450 microscope (FEI, Eindhoven, The Netherlands) under low vacuum conditions.

#### 2.4.5. Statistical Evaluation

In the statistical evaluation of the mechanical properties, 10 samples (N = 10) were used for each test. The results were analyzed for statistically significant differences (*p* < 0.05) using one-way ANOVA followed by Tukey’s multiple comparison test (GraphPad Prism, Version 5.03, GraphPad Software, San Diego, CA, USA).

## 3. Results and Discussion

### 3.1. Miscibility and Time Stability of Resins Blends and LR

Microscopic observation of both the unmodified resin blends (F and C) showed complete homogeneity, without the presence of inclusions or foreign phases that could cause light scattering and image formation. Polymerization also did not change the morphology of both resin blends.

**Figure 2:**
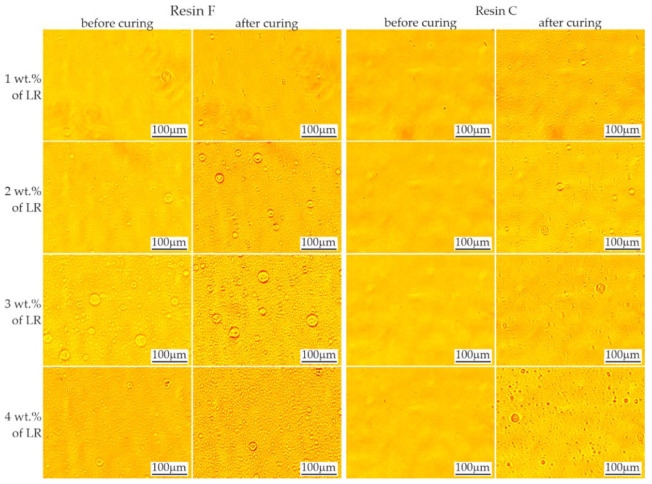
Evaluation of rubber miscibility in resins F and C. Rubber domains are visible as round objects.

The evaluation of the rubber solubility is shown in Figure 2. In the case of resin C with a lower viscosity, addition of LR in the range of 1–4 wt.%. produced no visible domains before the polymerization process. The domains were revealed after the process as fine spherical particles. On the other hand, resin F, which has a higher viscosity, did not tend to dissolve the rubber. The presence of rubber domains in this resin was noticeable even with 1 wt.% LR. Since only the BisGMA/TEGDMA resin ratio was changed in the blend, it can be assumed that the LR was dissolved by the lower viscosity resin (TEGDMA). Increasing the viscosity and molecular weight as a result of initiating the polymerization process altered the solubility conditions of the liquid rubber, causing the appearance of second phase precipitations—the LR domains.

**Figure 3:**
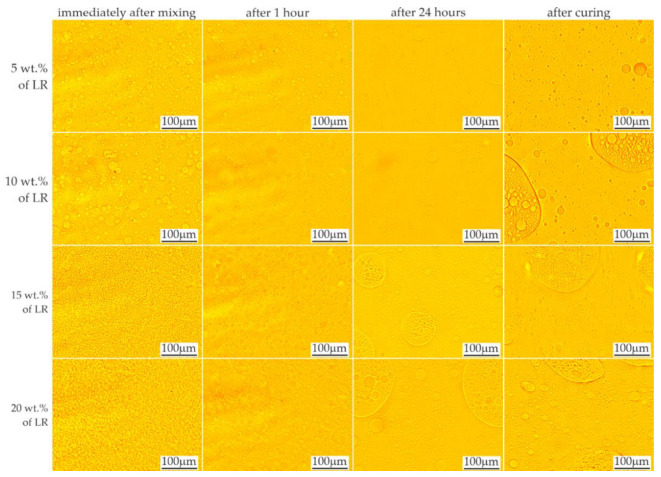
LOM images showing time stability of resin F mixed with liquid rubber.

**Figure 4:**
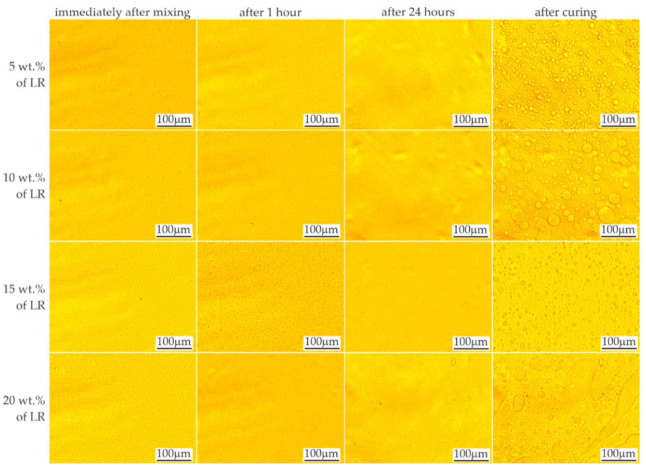
LOM images showing time stability of resin C mixed with liquid rubber.

The effects of mixing the resins with 5–20% by weight of liquid rubber and the time stability of the mixtures are shown in Figure 3 and Figure 4, respectively. When the LR concentration was increased up to 5 wt.%, domains with diameters ranging from less than 1 μm to approx. 50 μm were observed. With increased LR content, numerous domains were observed, even immediately after mixing, in accordance with the equilibrium system for the two-component polymer system [25]. Based on microscopic observations, the ultimate solubility in the C resin at ambient temperature can be determined as approx. 5%. Above this concentration, the presence of a second phase was observed, the amount of which increased with time, possibly due to the mixing shear forces dissapear. After 24 h, no changes in the size or morphology of the rubber domains were observed in the tested materials.

A video sequence showing the polymerization process was also recorded to assess the changes and kinetics of the process. The recorded sequences made it possible to observe the movement of the domains as the material spread over the slide surface. The beginning of polymerization process caused the movement to stop within approx. 2–4 s by creating a rigid structure that limited displacement. This period corresponds well with the dynamics of the changes observed by Ellakwa et al. [26] during the polymerization.

**Figure 5:**
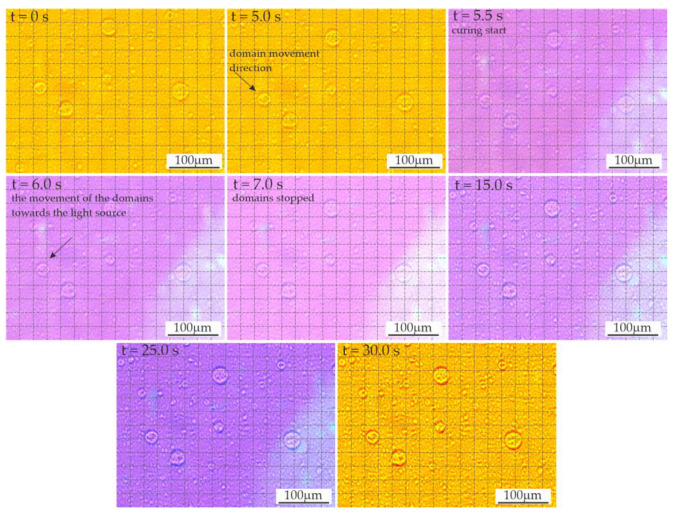
The dynamics of polymerization in the video sequence recorded for resin F containing 3 wt.% liquid rubber.

Approximately 5.5 s after application of the material on the slide glass, the photopolymerization was initiated (Figure 5) using a LED lamp. The first three images show the domains moving during this time (right and down direction). The grid applied to the images facilitated the comparison of the position of individual objects. The direction of the domains’ movement during polymerization was initially unchanged; then, after about 1 s, it changed, with the domains pointing towards the light source. After another second, the movement stopped. In the further period of polymerization of the resins modified with LR, phase separation was observed due to changes in viscosity and solubility. The effect of these changes appeared as enhancements of the domain edges due to changes in the optical properties.

The miscibility of the resins and liquid rubber at a specified temperature depends on many factors, including the function number and molecular weight of the monomer, and the molecular weight and dispersion of the LR. The mixture of resin and liquid rubber showed behavior following the equilibrium system [25]. As the curing proceeded, phase separation was induced by an increase in the molecular weight of the resin as the conversion progressed. Depending on the initial composition and curing conditions, phase separation occurred through a spinodal mechanism or a nucleation and growth mechanism [27].

### 3.2. Mechanical Properties

**Figure 6:**
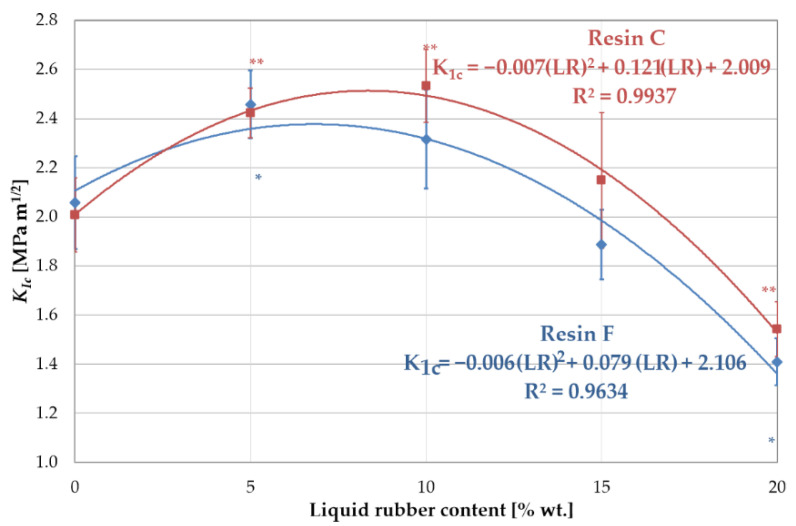
Fracture toughness *K_Ic_* of resin blends modified with liquid rubber. Symbols (*) and (**) indicates statistically significant differences in relation to the unmodified resin, for resin F and C, respectively.

**Figure 7:**
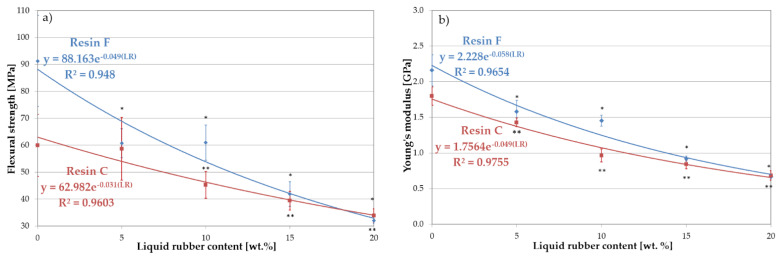
Mechanical properties of resin blends modified with liquid rubber: (**a**) flexural strength and (**b**) Young’s modulus. The symbols (*) and (**) indicate statistically significant differences in relation to the base resin, for resin F and C, respectively.

The results showed a significant increase in the *K_Ic_* value when 5 wt.% liquid rubber was added to resin F. Further increasing of the amount of LR weakened the toughening effect (Figure 6). The effect of viscosity (resulting directly from the proportions of BisGMA and TEGDMA) of resin F was evident when considering the fracture toughness with the miscibility. The high viscosity caused a reduced miscibility, as a result of which the domains of LR formed a discrete phase of relatively large dimensions in the matrix, especially for higher LR concentrations. The lack of proper integration of the LR with the resin blend resulted in a weakening of the bonds and, as a consequence, in the reduction of the fracture toughness. This is also confirmed by the values of flexural strength and Young’s modulus (Figure 7).

In the case of resin C, a significant increase in fracture toughness was observed with increasing concentrations of LR up to 10 wt.%. A further increase in the amount of LR resulted in a decrease in fracture toughness. Analogous considerations regarding viscosity and miscibility lead to the conclusion that the LR is better integrated with resin C. However, the presence of the low-viscosity component in this resin caused a significant reduction in both mechanical strength and Young’s modulus, compared to resin F (Figure 7). For higher liquid rubber concentrations, no increase in fracture toughness was found. This can be attributed to the larger size of the LR domains at the higher concentrations, as observed in the microscopy studies. In addition, the distribution and size of the domains changed. The large domains generated with high LR content attenuated the fracture toughness as a result of the high intensity of stresses around the agglomerated domains of the LR. The improvement in fracture toughness, as explained by Kinloch and Hunston [28], can be attributed to the LR phase, which concentrated shear stresses in a specific area, acting as stress concentrators. The hydrostatic pressure in front of the crack tip causes the domains to rapidly cavitate. Consequently, the cavitated failure zone dulled the crack tip, which behaved as if it had a much larger tip radius.

Thus, a larger plastic deformation zone was associated with the crack, which was the source of the toughening effect. The interfacial interactions of the domains with the resin were also desirable as they allowed the miscibility to be increased, and the partial reaction of the rubber with the matrix resulted in improved strength and fracture toughness. A certain amount of LR also acted as a plasticizer if the LR could react with the resin. Hypro 2000X168 VTB rubber is able to undergo such reaction via functionalized vinyl-chain ends [29]. Both of these effects increased the shear deformation capacity of the resin. However, since the LR did not have significant miscibility with the resin, the plasticization of the matrix was low and only the elasticizing effect was present. This was likely the reason for a slight increase in the *K_Ic_* value when increasing the rubber concentration.

Kerby et al. [10] obtained over 4-times lower *K_Ic_* values for the BisGMA/TEGDMA resin mixture (50/50% by weight) after modification with liquid rubber. Differences in the research methodology adopted should be noted here; moreover, a large amount of TEGDMA resin with a very low viscosity (0.01 Pa·s) was used. Interestingly, in the case of resin C (low viscosity), a similar trend of changes in the *K_Ic_* value was obtained compared to the work in [10].

An exponential decrease in flexural strength was observed with increasing LR concentration (Figure 7a). For the unmodified resins, this strength was 91 MPa for resin F and 64 MPa for resin C. The influence of the lower viscosity of resin C on the strength value was clearly visible here. Increasing the liquid rubber content to 5 wt.%. resulted in a reduction in strength to 70 MPa and 62 MPa for resins F and C, respectively. The presence of LR reduced the stiffness of the resin network, possibly due to a decrease in the crosslink density. The insolubility and low compatibility of LR with resin F, and the percentage of the dispersed rubber phase was more significant than resin C.

Both types of unmodified resins exhibited an elastic character under bending, with Young’s modulus values of 2.16 GPa and 1.79 GPa for resins F and C, respectively (Figure 7b). The introduction of liquid rubber caused a decrease in the value of Young’s modulus. Simultaneously, a plastic characteristic of the materials appeared. It was confirmed by the fact that most of the specimens with 15% or 20% LR were not completely fractured. Thomas et al. [8] observed a similar phenomenon in their studies of epoxy resin strengthened with the CTBN modifier.

Increasing the amount of LR to 5 wt.% led to a reduction in Young’s modulus to 1.58 GPa for resin F and 1.43 GPa for resin C (27% and 21%, respectively), while at 10 wt.% LR, this value drops to 1.45 GPa and 0.96 GPa (33% and 46%, respectively) for resins F and C, respectively. This lower value of Young’s modulus for both modified resins could be attributed to a decrease in network cross-link density. Moreover, according to [25], the location of the modifier in the reaction centers during polymerization could also lower the stiffness of the network.

### 3.3. Microscopic Evaluation of the Fractures

**Figure 8:**
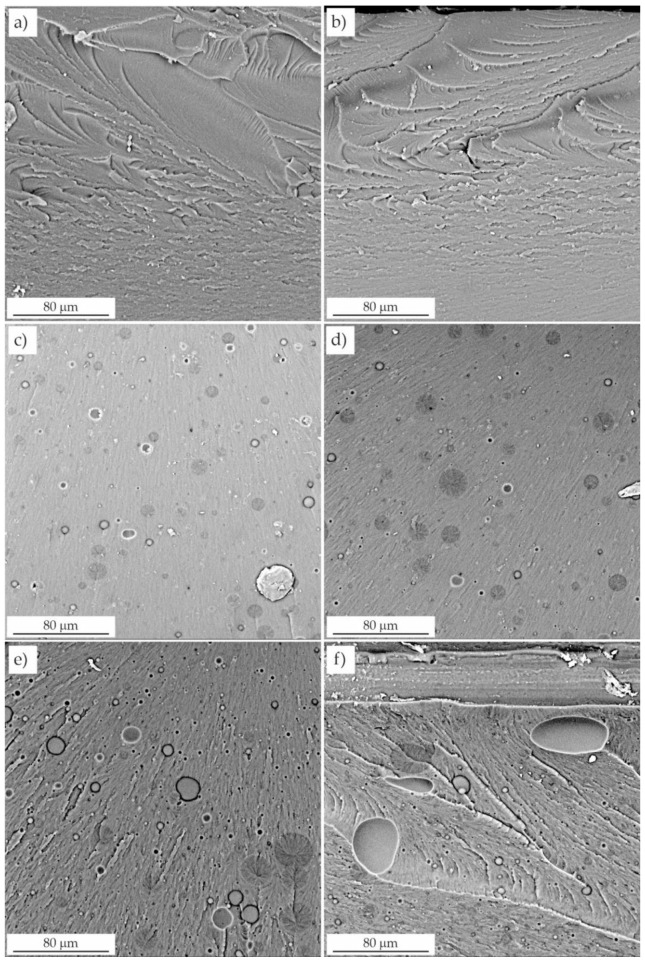
Fractures of unmodified and modified resins. (**a**) resin F, (**b**) resin C, (**c**) resin C + 5 wt.% LR, (**d**) resin C + 5 wt.% LR, (**e**) resin F + 10 wt.% LR, (**f**) resin C + 10 wt.% LR.

The fracture surfaces were observed by scanning electron microscopy. Unmodified resins F and C (Figure 8) presented smooth and glassy surfaces with grooves and waves. A smooth fracture area, regardless of the presence of shear deformation lines, indicated that no significant plastic deformation occurred. Waves were caused by brittle a fracture, as there were no energy dissipation mechanisms.

**Figure 9:**
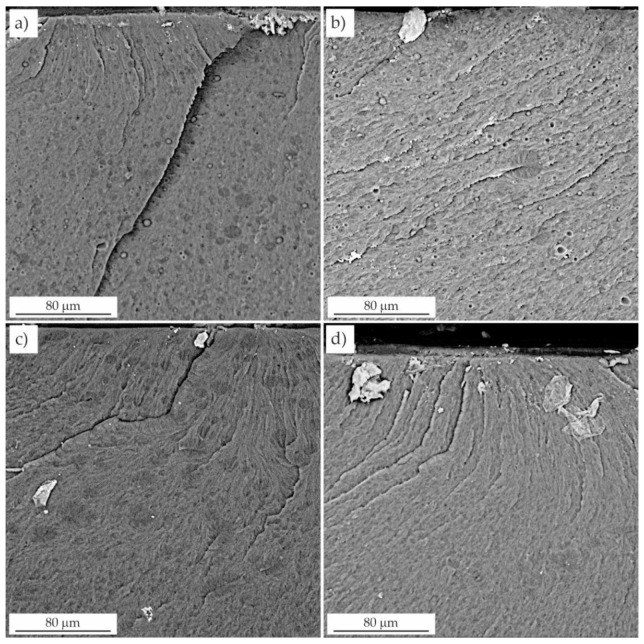
Fractures of resins modified with liquid rubber. (**a**) resin F + 15 wt.% LR, (**b**) resin C + 15 wt.% LR, (**c**) resin F + 20 wt.% LR, (**d**) resin C + 20 wt.% LR.

Two distinct phases were clearly visible on the fractures of the modified resins (Figure 8 and Figure 9): the continuous resin matrix and the dispersed LR phase. This morphology made the surface dull and increased its roughness. Smaller domains and craters were visible as residues, especially of larger particles torn from the surface during fracture. Remnants of the rubber phase were observed in these craters.

In contrast to the unmodified resin, resins toughened with LR exhibited microplasticity in the form of elongated cavities. Pearson and Yee [30] showed that rubber was present inside the cavitation voids, suggesting that the domains cavitated internally. The surfaces of the voids had a texture that was likely due to the residual LR phase.

SEM analysis also revealed the presence of a whitened zone due to microcavitation of the rubber domains as a result of high hydrostatic stress near the crack tip. The presence of small, densely spaced voids was also observed, which was due to the expanding deformation of the domains and the resin [31], which initiated local plastic deformation of the resin and significant crack tip deflection. The fracture toughness of the resins modified with liquid rubber resulted from the energy dissipation occurring near the crack tip. The synergistic effects of cavitation located at the LR/resin interface and plastic shear in the resin were presumably responsible for the deformation that dissipated the energy. The effect of this dissipation was the improvement of the fracture toughness values.

Increasing the concentration of liquid rubber in the resin resulted in the formation of numerous domains of various sizes. The failure began with the formation of microcracks, which were also present at lower LR concentrations, but their number was significantly smaller. They occurred mainly in the end fracture zone, where the domains facilitated the propagation of the crack by the notch, and the value of the failure stress increased significantly. This was in line with Griffith’s theory of the void size initiating fracture for a certain stress level.

Microscopic observations showed that both the number and the average diameter of the domains increased with LR concentration. This is consistent with the behavior of other resins modified in this way [25]. The increase in domain size with increasing rubber concentration was attributed to the coalescence of the dispersed regions of the LR, which depended on their viscosity and elasticity. In the case of resins modified with 5 wt.% and 10 wt.% LR, the domains were evenly distributed throughout the resin matrix and have a relatively low particle size range (Figure 8). The morphology of the smaller domains was responsible for the reduced crack propagation in these samples, as indicated by the presence of a relatively large number of deformation lines. Moreover, the fractures were not smooth (Figure 8) in contrast to the unmodified resin. According to Yee and Pearson [30], the size of the whitening zone (due to crazing), and the number of deformation lines is proportional to the increase in the fracture toughness. Deformation of the LR domains in the cured resin was attributed to greater plastic deformation. The deformation lines propagated through the LR domains, which promoted stress transfer between the particles and the resin matrix. Furthermore, whitening of the interfacial layer around the LR domains indicated an interaction between the domains and the matrix in samples with a lower LR content [32]. In turn, Lee et al. [33] and Bascom and Hunston [31] found that the stress whitening was caused by the dispersion of visible light on the scattering center layers, which were voids formed in the matrix as a result of LR cavitation. This was one of the most important energy dissipation mechanisms present in the modified resins.

### 3.4. Optimizing the Amount of LR

Introducing liquid rubber into dental composites produces (to a certain extent) an increase in the fracture toughness and a continuous exponential decrease in the Young’s modulus and flexural strength. However, the value of the flexural strength of the composites meets the requirements of the ISO 4049 standard [6] while the value of the E modulus decreased significantly, with a substantial difference in relation to the E modulus of tooth tissues (10–15 GPa for dentin and 20–80 GPa for enamel [34]). The composites showed an E value of 5–9 GPa [6]. Lowering this value will increase the property mismatch. Thus, the determination of the optimal amount of rubber was based on the relationship *K_Ic_* = f(*E*).

**Figure 10:**
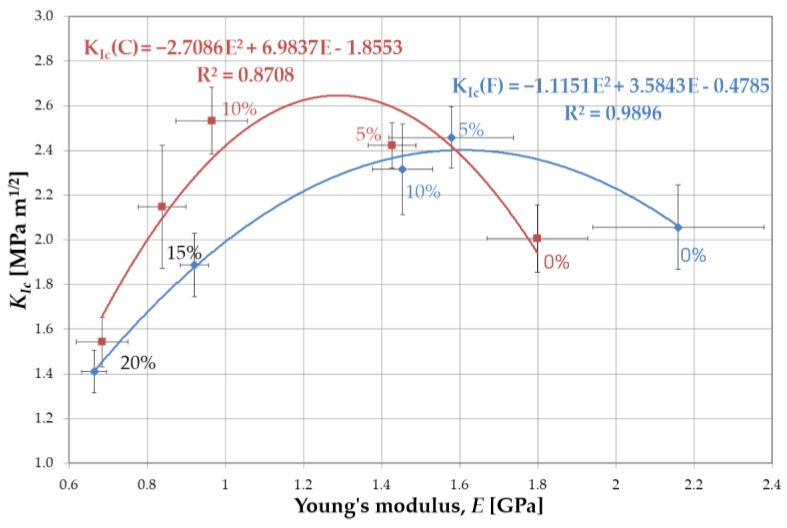
Optimization of the concentration of rubber in resins based on the analysis of fracture toughness and Young’s modulus.

The obtained results made it possible to the develop the relationship *K_Ic_* = f(*E*) for resins F and C (Figure 10). The data was approximated with the 2nd degree polynomial, which determined the maximum value of fracture toughness. This, in turn, made it possible to define the optimal concentration of liquid rubber.

For resin F, the functional relationship *K_Ic_* = f(*E*) is given by the equation:

*K_Ic_* = −1.1151*E*^2^
*+* 3.5843*E* − 0.4785 (4)

the maximum of which occurs at *E* = 1.63 GPa. Hence, from the exponential curve equation (Figure 7b):

*E* = 2228 e^−0.058(LR%)^ (5)

the optimal liquid rubber content in resin F is 5.4 wt.%.

For resin C, the relation between fracture toughness and the Young’s modulus is described by formula:

*K_Ic_* = −2.71*E*^2^
*+* 6.98*E* − 1.8553 (6)

the maximum of which occurs at *E* = 1.17 GPa. Hence, from the exponential curve equation (Figure 7b):

*E* = 1756.4 e^−0.049(LR%)^ (7)

the optimal liquid rubber content in resin C was determined to be 8.3 wt.%.

For resin F, an increase in fracture toughness was observed at 5% by weight of LR, while the strength and Young’s modulus were reduced by 23% and 27%, respectively. For resin C, an increase in fracture toughness up to 10 wt.% LR was observed, while the reduction in strength was 25% and 28% for 5% and 10% rubber, respectively. For Young’s modulus, these reductions were even higher and were 21% and 46%, respectively. It is noteworthy that as the solubility increases, so does the optimal amount of liquid rubber.

## 4. Conclusions

The null hypothesis of no difference between resin blends without and after modification with liquid rubber has been rejected. The results of the tests showed that the ratio of the BisGMA/TEGDMA resins influenced the solubility of the liquid rubber. The higher content of the TEGDMA resin (40 wt.%) resulted in LR solubility at the level of 5%. However, a 20 wt.% concentration of TEGDMA in the mixture caused the liquid rubber to be almost insoluble. Resin blends containing LR showed good time stability. Such mixtures showed no changes in the size or morphology of the domains 24 h after mixing. The maximum value of fracture toughness (2.46 MPa m1/2) was obtained for resin F containing 5 wt.% LR, while for resin C it was obtained for 15 wt.% LR (2.53 MPa m1/2). Exponential reductions in both the flexural strength and Young’s modulus were observed after modification with LR. The obtained results made it possible to determine the optimal LR content for both blends. Specifically, for resin F it was 5.4 wt.%, while for the resin C it was 8.3 wt.%. Increasing the solubility of the LR in the blend also increases its optimal content.

## Data Availability

Data available upon request.

## References

[1] Ferracane J.L. (2011). Resin Composite—State of the Art. Dent. Mater..

[2] Bagheri R., Marouf B.T., Pearson R.A. (2009). Rubber-Toughened Epoxies: A Critical Review. Polym. Rev..

[3] Schwass D.R., Lyons K.M., Purton D.G. (2013). How Long Will It Last? The Expected Longevity of Prosthodontic and Restorative Treatment. N. Z. Dent. J..

[4] Da Rosa Rodolpho P.A., Donassollo T.A., Cenci M.S., Loguércio A.D., Moraes R.R., Bronkhorst E.M., Opdam N.J.M., Demarco F.F. (2011). 22-Year Clinical Evaluation of the Performance of Two Posterior Composites with Different Filler Characteristics. Dent. Mater..

[5] Pałka K. (2020). Polymerization Shrinkage of New Dental Composites Modified with Liquid Rubber. Eng. Biomater..

[6] Palka K., Kleczewska J., Sasimowski E., Belcarz A., Przekora A. (2020). Improved Fracture Toughness and Conversion Degree of Resin-Based Dental Composites after Modification with Liquid Rubber. Materials.

[7] Pałka K., Miazga-Karska M., Pawłat J., Kleczewska J., Przekora A. (2021). The Effect of Liquid Rubber Addition on the Physicochemical Properties, Cytotoxicity and Ability to Inhibit Biofilm Formation of Dental Composites. Materials.

[8] Thomas R., Abraham J., Thomas P.S., Thomas S. (2004). Influence of Carboxyl-Terminated (Butadiene-Co-Acrylonitrile) Loading on the Mechanical and Thermal Properties of Cured Epoxy Blends. J. Polym. Sci. B Polym. Phys..

[9] Kong J., Ning R., Tang Y. (2006). Study on Modification of Epoxy Resins with Acrylate Liquid Rubber Containing Pendant Epoxy Groups. J. Mater. Sci..

[10] Kerby R.E., Tiba A., Knobloch L.A., Schricker S.R., Tiba O. (2003). Fracture Toughness of Modified Dental Resin Systems. J. Oral. Rehabil..

[11] Garg A.C., Mai Y.W. (1988). Failure Mechanisms in Toughened Epoxy Resins-A Review. Compos. Sci. Technol..

[12] Sultan J.N., Liable R.C., McGarry F.J. (1971). Microstructure of Two-Phase Polymers. Polym. Symp..

[13] Ligon-Auer S.C., Schwentenwein M., Gorsche C., Stampfl J., Liska R. (2016). Toughening of Photo-Curable Polymer Networks: A Review. Polym. Chem..

[14] Cole P., Mandel J.S., Collins J.J. (2008). Acrylonitrile and Cancer: A Review of the Epidemiology. Regul. Toxicol. Pharmacol..

[15] Rodford R.A. (1990). Further Development and Evaluation of High Impact Strength Denture Base Materials. J. Dent..

[16] Rodford R.A., Braden M. (1992). Further Observations on High Impact Strength Denture-Base Materials. Biomaterials.

[17] Rodford R. (1986). The Development of High Impact Strength Denture-Base Materials. J. Dent..

[18] Teshima H., Matsukawa S. (1990). A Study on the Improvement of Denture Base Resin. Epoxy Dimethacrylate-Polybutadiene Dimethacrylate-MMA Monomers as the Liquid of Denture Base Resin. Shika Zair. Kikai.

[19] Cornell J.A. (1969). Impact Resistant, Alkali-Washed Mixed Butadiene-Styrene and Methyl Methacrylate Molding Composition. US Patent.

[20] Pałka K., Kleczewska J., Kalbarczyk G. (2021). Light-Curing Dental Composite Modified with Liquid Rubber and the Method for Its Production. Pat. Off. Repub. Pol..

[21] (2020). Standard Test Method for Linear-Elastic Plane-Strain Fracture Toughness of Metallic Materials.

[22] Ilie N., Hickel R., Valceanu A.S., Huth K.C. (2012). Fracture Toughness of Dental Restorative Materials. Clin. Oral. Investig..

[23] Fujishima A., Ferracane J.L. (1996). Comparison of Four Modes of Fracture Toughness Testing for Dental Composites. Dent. Mater..

[24] (2019). Dentistry—Polymer-Based Restorative Materials.

[25] Thomas R., Yumei D., Yuelong H., Le Y., Moldenaers P., Weimin Y., Czigany T., Thomas S. (2008). Miscibility, Morphology, Thermal, and Mechanical Properties of a DGEBA Based Epoxy Resin Toughened with a Liquid Rubber. Polymer.

[26] Ellakwa A., Cho N., Lee I.B. (2007). The Effect of Resin Matrix Composition on the Polymerization Shrinkage and Rheological Properties of Experimental Dental Composites. Dent. Mater..

[27] Chen J.P., Lee Y. (1995). Der A Real-Time Study of the Phase-Separation Process during Polymerization of Rubber-Modified Epoxy. Polymer.

[28] Kinloch A.J., Hunston D.L. (1986). Effect of Volume Fraction of Dispersed Rubbery Phase on the Toughness of Rubber-Toughened Epoxy Polymers. J. Mater. Sci. Lett..

[29] Huntsman International LLC USA Hypro® 2000X168LC VTB (2021). Technical Bulletin. www.Huntsman.Com/Products/Detail/524/Hypro.

[30] Yee A.F., Pearson R.A. (1986). Toughening Mechanisms in Elastomer-Modified Epoxies. Part 1: Mechanical Studies. J. Mater. Sci..

[31] Bascom W.D., Hunston D.L., Riew C.K. (1989). Fracture of Elastomer-Modified Epoxy Polymers. Rubber Toughened Plastics.

[32] Ozturk A., Kaynak C., Tincer T. (2001). Effects of Liquid Rubber Modification on the Behaviour of Epoxy Resin. Eur. Polym. J..

[33] Lee W.H., Hodd K.A., Wright W.W., Riew C.K. (1989). Phase-Separation and Transition Phenomena in Toughened Epoxies. Rubber Toughened Plastics.

[34] Gradl R., Zanette I., Ruiz-Yaniz M., Dierolf M., Rack A., Zaslansky P., Pfeiffer F. (2016). Mass Density Measurement of Mineralized Tissue with Grating-Based X-Ray Phase Tomography. PLoS ONE.

